# Evaluation of Potential Effects of Increased Outdoor Temperatures Due to Global Warming on Cerebral Blood Flow Rate and Respiratory Function in Chronic Obstructive Disease and Anemia

**DOI:** 10.1002/gch2.202300120

**Published:** 2023-09-12

**Authors:** Surhan Bozkurt, Selim Bozkurt

**Affiliations:** ^1^ Department of Electrical and Electronics Engineering Dogus University Esenkent Dudullu OSB m. NATO Yolu c. Umraniye Istanbul 34775 Turkey; ^2^ School of Engineering Ulster University 2–24 York Street Belfast BT15 1AP UK

**Keywords:** anemia, cerebral blood flow, COPD, global warming, public health, respiratory function

## Abstract

Global warming due to increased outdoor carbon dioxide (CO_2_) levels may cause several health problems such as headaches, cognitive impairment, or kidney dysfunction. It is predicted that further increases in CO_2_ levels will increase the morbidity and mortality of patients affected by a variety of diseases. For instance, patients with Chronic Obstructive Pulmonary Disease (COPD) may suffer cognitive impairments or intracranial bleeding due to an increased cerebral blood flow rate. Predicting the harmful effects of global warming on human health will help to take measures for potential problems. Therefore, the quantification of physiological parameters is an essential step to investigate the effects of global warming on human health. In this study, the effects of increased outdoor temperatures due to climate change on cerebral blood flow rate and respiratory function in healthy subjects and COPD patients with anemia and respiratory acidosis are evaluated utilizing numerical simulations. The numerical model simulates cardiac function and blood circulation in systemic, pulmonary and cerebral circulations, cerebral autoregulatory functions, respiratory function, alveolar gas exchange, oxygen (O_2_) and CO_2_ contents, and hemoglobin levels in the blood. The simulation results show that although the cardiovascular function is not significantly altered, the respiratory function and cerebral blood flow rates are altered remarkably.

## Graphical abstract

Predicting the harmful effects of global warming on human health will help to take measures for potential problems. Therefore, the quantification of physiological parameters is an essential step to investigate the effects of global warming on human health. Effects of the increased outdoor temperatures on the cerebral circulation in healthy subjects and COPD patients with anemia are simulated using a computational model.

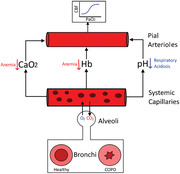

## 1 Introduction

Global warming is a result of increased outdoor carbon dioxide (CO_2_) levels due to fossil fuel combustion.^[^ ^1^ ^]^ The level of outdoor CO_2_ was around 280 ppm in the preindustrial period whereas it increased to around 400 ppm at present.^[^ ^1^, ^2^ ^]^ The climate models show that the outdoor CO_2_ level may increase to around 1000 ppm by 2100 and around 2000 ppm by 2250.^[^ ^2^, ^3^ ^]^ The indoor CO_2_ level in crowded areas may increase up to ten times depending on the outdoor CO_2_ level.^[^ ^4^, ^5^ ^]^ Breathing more CO_2_ leads to harmful effects on physiology such as headaches, dizziness, wheezing, and eye irritation.^[^ ^6^, ^7^ ^]^ Also, cognitive impairment,^[^ ^8^ ^]^ kidney calcification,^[^ ^9^ ^]^ and hypercapnia due to a decrease in blood pH^[^ ^10^ ^]^ are associated with breathing more CO_2_. Furthermore, poor air quality leads to the death of 1.6 million people annually, especially in countries more affected by global warming due to various health problems associated with elevated CO_2_.^[^ ^11^ ^]^

Chronic obstructive pulmonary disease (COPD) is a respiratory disease caused by narrowed respiratory airways.^[^ ^12^ ^]^ The partial pressure of arterial CO_2_ is higher in patients with COPD than in healthy subjects^[^ ^13^ ^]^ and the level of inspired CO_2_ affects the partial pressure of arterial CO_2_.^[^ ^14^ ^]^ Three million people die due to COPD each year^[^ ^15^ ^]^ and it is predicted that COPD will be the third leading disease causing death around 2030.^[^ ^15^ ^]^ It is also predicted that global warming will have negative effects on respiratory and neurological diseases because poor air quality due to global warming may impair lung function,^[^ ^16^, ^17^ ^]^ and increase morbidity and mortality in patients with COPD.^[^ ^18^ ^]^

There is an interaction between the respiratory system and cerebral blood flow rate.^[^ ^19^ ^]^ An increase in the partial pressure of arterial CO_2_^[^ ^20^ ^]^ and the reduced arterial oxygen (O_2_) content due to a decrease in hemoglobin level cause the cerebral blood flow rate to increase.^[^ ^20^, ^21^ ^]^ The higher cerebral blood flow rate may cause cognitive impairments,^[^ ^5^ ^]^ intracranial bleeding, and stroke.^[^ ^22^ ^]^ Therefore, it is thought that global warming may increase deaths related to neurodegenerative disorders.^[^ ^23^, ^24^ ^]^ Predicting the harmful effects of global warming on human health will help to take measures for the potential problems and different studies in the literature try to predict the potential effects of global warming on human health.^[^ ^25^, ^26^ ^]^ Therefore, the quantification of physiological parameters is an essential step to investigate the effects of global warming on human health. Computational simulations have been used in a wide range of applications to predict the outcome of the physiological cases and therapies used in clinics.^[^ ^27^, ^28^ ^]^ They have also the potential to evaluate and predict the effects of global warming on physiological systems and human health.

In this study, the effects of acidosis which is one of the results of the increased outdoor temperatures and CO_2_ levels on the cerebral blood flow rate and respiratory function in healthy subjects and COPD patients with anemia were evaluated utilizing numerical simulations.

## 2 Experimental Section

**Figure 1:**
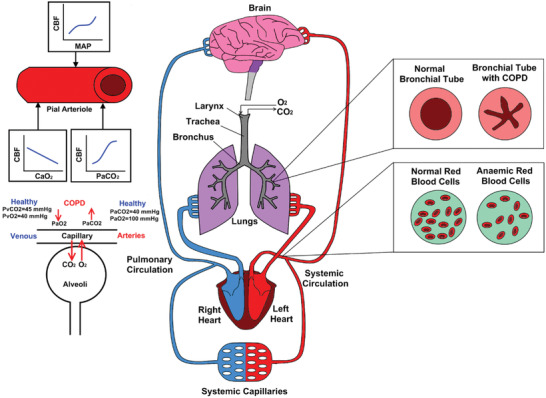
Diagram of the heart, lung, brain, and circulatory system for healthy and COPD conditions (**COPD**: Chronic Obstructive Pulmonary Disease, **MAP**: Mean Arterial Pressure, **CBF**: Cerebral Blood Flow Rate).

Effects of the increased outdoor temperatures on the cerebral circulation in healthy subjects and COPD patients with anemia were simulated using a computational model which describes blood flow rates and pressures in cardiovascular system and cerebral circulation, cerebral autoregulatory and systemic arteriolar baroreflex regulation, air flow rates and pressures in respiratory system, gas exchange mechanism between alveoli and blood and arterial and vein blood O_2_ and CO_2_ contents. The simulations were performed using Matlab Simulink 2021b. The ode15s solver was used to solve the set of equations whereas the maximum step size and relative tolerance were 0.001 and 1e‐3. Simulated physiological systems and mechanisms causing COPD and anemia are illustrated in **Figure** **1**.

### 2.1 Cardiac Function and Blood Circulation in the Cardiovascular System

Left ventricular pressure (*p_lv_*) was modeled using active and passive contractions (*p_lv,a_, p_lv,p_*) over the systolic and diastolic phases. Active contraction of the left ventricle (*p_lv,a_*) was modeled using left ventricular end‐systolic elastance (*E_es,lv_*), left ventricular volume (*V_lv_*), zero‐pressure volume (*V_lv,0_*) and activation function of the left ventricle (*f_act,lv_*). Passive contraction (*p_lv,p_*) of the left ventricle was modeled using left ventricular volume and zero‐pressure volume.

(1) $$p_{lv}=p_{lv,a}+p_{lv,p}$$

(2) $$p_{lv,a}\left(t\right)=E_{es,lv}\left(V_{lv}-V_{lv,0}\right)f_{act,lv}$$

(3) $$p_{lv,p}=A\left(e^{B\left(V_{lv}-V_{lv,0}\right)}-1\right)$$

Here, *A* and *B* are constants in the left ventricular passive pressure model. Left ventricular volume was modeled using the radius and long axis length of the left ventricle (*r_lv_*, *l_lv_*).^[^ ^29^ ^]^

(4) $$V_{lv}=\frac{2}{3}\pi K_{lv}r_{lv}^{2}l_{lv}$$

*K_lv_* is a coefficient that includes the effects of the left ventricular contraction in the long axis. Change of the left ventricular radius (*dr_lv_/dt*) was modeled using blood flow rates of mitral and aortic valves (*Q_mi_*, *Q_av_*), left ventricular volume (*V_lv_*), *K_lv_* and long axis length (*l_lv_*).^[^ ^29^ ^]^

(5) $$\frac{dr_{lv}}{dt}=\frac{3\left(Q_{mv}-Q_{av}\right)}{4\pi K_{lv}l_{lv}}{\left(\frac{3V_{lv}}{2\pi K_{lv}l_{lv}}\right)}^{-1/2}$$

Left atrial pressure (*p_la_*) was modeled using left atrial elastance (*E_la_*), radius and long axis length of the left atrium (*r_la_*, *l_la_*), the radius at zero‐pressure volume (*r_la,0_*) and a scaling coefficient (*K_la_*). Left atrial volume (*V_la_*) was modeled using the radius and long‐axis length of the left atrium (*r_la_*, *l_la_*) and a scaling coefficient (*K_la_*).^[^ ^29^ ^]^ Left atrial pressure and volume are given in equations (6) and (7).

(6) $$p_{la}=E_{la}\left(t\right)\left[\frac{4}{6}\pi K_{la}l_{la}\left(r_{la}^{2}-r_{la,0}^{2}\right)\right]$$

(7) $$V_{la}=\frac{4}{6}\pi K_{la}l_{la}r_{la}^{2}$$

Left atrial elastance (*E_la_*) was modeled using maximum and minimum elastance of the left atrium (*E_la,max_*, *E_la,min_*) and left atrial activation function (*f_act,la_*).^[^ ^30^ ^]^

(8) $$E_{la}\left(t\right)=E_{la,\min}+0.5\left(E_{la,\max}-E_{la,\min}\right)f_{act,la}\left(t-D\right)$$

Change of the left atrial radius (*dr_la_/dt*) was modeled using blood flow rates through the pulmonary veins (*Q_vp_*) and mitral valve (*Q_mv_*), a scaling coefficient (*K_la_*), left atrial long‐axis length (*l_la_*) and left atrial volume (*V_la_*).^[^ ^29^ ^]^

(9) $$\frac{dr_{la}}{dt}=\frac{3\left(Q_{pv}-Q_{mv}\right)}{4\pi K_{la}l_{la}}{\left(\frac{3V_{la}}{2\pi K_{la}l_{la}}\right)}^{-1/2}$$

Right ventricular and atrial pressure and volume were modeled in a similar way using different parameters. Heart valves were modeled as ideal diodes permitting a unidirectional flow rate. Detailed information about the models for right heart chambers was given in Bozkurt 2019.^[^ ^29^ ^]^

Change of the aortic pressure (*dp_ao_/dt*) was modeled using aortic compliance (*C_ao_*), blood flow rates through the aortic valve (*Q_av_*) and aorta (*Q_ao_*) over a cardiac cycle. Change of blood flow rate in the aorta with respect to time (*dQ_ao_/dt*) was modeled using aortic resistance (*R_ao_*) aortic inertance (*L_ao_*), aortic pressure (*p_ao_*), pressure in the aortic arch (*p_aa_*) and blood flow rate through the aorta (*Q_ao_*).

(10) $$\frac{dp_{ao}}{dt}=\frac{Q_{av}-Q_{ao}}{C_{ao}}$$

(11) $$\frac{dQ_{ao}}{dt}=\frac{p_{ao}-p_{aa}-Q_{ao}R_{ao}}{L_{ao}}$$

Blood flow rates and pressures in the other compartments were modeled in a similar way using different parameters.

The cerebral circulation model includes left and right internal carotid arteries, left and right vertebral arteries, basilar artery, left and right anterior cerebral arteries, left and right middle cerebral arteries, left and right posterior cerebral arteries, left and right anterior choroidal arteries, superior cerebellar arteries, left and right posterior communicating arteries, anterior communicating artery, left and right ophthalmic arteries, pial arterioles, cerebral capillaries and cerebral veins.^[^ ^31^ ^]^

### 2.2 Autoregulatory Mechanisms

#### 2.2.1 Systemic Arteriolar Resistance Regulation

Systemic arteriolar resistance (*R_ars_*) was tuned depending on mean aortic pressure (*p_ao,m_*).^[^ ^32^ ^]^

(12) $$\Delta R_{ars}=\left|S_{Rars}\left(p_{ao,ars,set}-p_{ao,m}\right)R_{ars,set}\right|$$

(13) $$R_{ars}=\left\{\begin{array}{c} R_{ars}-\Delta R_{ars}\;p_{ao,m}>p_{ao,ars,set}\\ R_{ars}+\Delta R_{ars}\;p_{ao,m}\leq p_{ao,ars,set} \end{array}\right.$$

In the equations above, *∆R_ars_*, *R_ars,set_*, *p_ao,ars,set_* and *S_ars_* are change in the systemic arteriolar resistance, systemic arteriolar resistance at aortic pressure set point, aortic pressure set point and sensitivity of the systemic arteriolar resistance, respectively.

#### 2.2.2 Pial Arteriolar Resistance Regulation

Pial arteriolar resistance (*R_pc_*) was described using interaction among cerebrovascular CO_2_ reactivity (*R_pc,pCO2_*), cerebrovascular O_2_ content (*R_pc,CO2_*) and static cerebral autoregulatory function (*R_pc,pao,m_*).^[^ ^31^ ^]^

(14) $$R_{pc}=R_{pc,set}\cdot R_{pc,p_{CO2}}\cdot R_{pc,c_{O2}}\cdot R_{pc,p_{ao,m}}$$

R_pc,set_ is a set point of pial arteriolar resistance for a healthy state.

#### 2.2.3 Cerebrovascular CO_2_ Reactivity

The increase in the partial pressure of arterial CO_2_ causes the cerebral blood flow rate to increase due to an increase in the radius of cerebral arteries.^[^ ^33^ ^]^ An exponential was used to describe the relationship between pial arteriolar resistance (*R_pc,CO2_*) and partial pressure of arterial CO_2_ (*p_a,CO2_*).^[^ ^31^ ^]^

(15) $$R_{pc,CO_{2}}=R_{pc,set}\cdot a_{1}\cdot e^{a_{2}\cdot p_{a,CO_{2}}}$$

*R_pc,set_* is a set value of the pial arteriolar resistance when the partial pressure of arterial CO_2_ (*p_aCO2_*) is 40 mmHg. *a_1_* and *a_2_* represent coefficients in the model.

#### 2.2.4 Arterial O_2_ Content

The decrease of O_2_ content in the arterial blood causes cerebral arteries to dilate and the cerebral blood flow rate increases. A linear relationship was used to describe pial arteriolar resistance (*R_pc,CaO2_*).

(16) $$R_{pc,C_{aO_{2}}}=R_{pc,set}\cdot \left(a\cdot C_{aO_{2}}+b\right)$$

*R_pc,set_* is the pial arteriolar resistance setpoint at physiological O_2_ content in the blood, *a* and *b* empirical coefficients in the model. Arterial O_2_ content was defined using hemoglobin concentration (*Hb*), arterial oxygen saturation (*S_aO2_*) and the partial pressure of arterial oxygen (*p_aO2_*).^[^ ^34^ ^]^

(17) $$C_{aO_{2}}=1.34\cdot Hb\cdot S_{aO_{2}}+p_{aO_{2}}0.0031$$

#### 2.2.5 Static Cerebral Blood Flow Rate Autoregulation

The cerebral blood flow rate almost remains constant while the aortic pressure is between 60 mmHg and 150 mmHg.^[^ ^35^ ^]^ Change of the pial arteriolar resistance was modeled using the sensitivity of pial arteriolar resistance (*S_Rpc,pao_*), pial arteriolar resistance at aortic pressure set point (*R_pc,set_*), aortic pressure set point (*p_ao,cbf,set_*) and mean aortic pressure (*p_ao,m_*).^[^ ^32^ ^]^

(18) $$\Delta R_{pc,pao}=\left|S_{Rpc,pao}\left(p_{ao,cbf,set}-p_{ao,m}\right)R_{pc,set}\right|$$

Pial arteriolar resistance (*R_pc,pao_*) was modeled using change of the pial arteriolar resistance (*∆R_pc,pao_*).

(19) $$R_{pc,pao}=\left\{\begin{array}{c} R_{pc,pao}+\Delta R_{pc,pao}\;p_{ao,m}>p_{ao,cbf,set}\\ R_{pc,pao}-\Delta R_{pc,pao}\;p_{ao,m}\leq p_{ao,cbf,set} \end{array}\right.$$

### 2.3 Respiratory System

Air flow rates and pressures during inspiration and expiration were modeled in the larynx, trachea, bronchia and alveoli using equivalent resistance (R) and compliance (C) elements.^[^ ^36^ ^]^ Breathing in and out was driven by an activation function describing pleural pressure over a respiratory cycle.^[^ ^36^ ^]^ Pleural pressure was modeled using the amplitude of pleural pressure (*p_pl,amp_*), instantaneous time (*t*), respiration period (*T*), inspiration time (*T_i_*), and expiration time (*T_e_*).

(20) $$p_{pl}\left(t\right)=\left\{\begin{array}{c} \frac{-p_{pl,amp}}{T_{i}\cdot T_{e}}\cdot t^{2}+\frac{p_{pl,amp}\cdot T}{T_{i}\cdot T_{e}}\cdot t-5\;0\leq t\leq T_{i}\\ \frac{p_{pl,amp}}{1-e^{-\frac{T_{e}}{\tau }}}\left(e^{-\left(\frac{t-T_{i}}{\tau }\right)}-e^{-\frac{T_{e}}{\tau }}\right)-5\;T_{i}<t\leq T \end{array}\right.$$

Here, τ is the time constant of the expiration process. Change of the pressure in the larynx (*dp_pl_/dt*) was modeled using atmospheric pressure (*p_atm_*), pressure in the larynx (*p_l_*) and pressure in the trachea (*p_tr_*). The inspired airflow rate (*Q_airl_*) was modeled using atmospheric pressure (*p_atm_*), and pressure in the larynx (*p_l_*). Air volume in the larynx (*V_l_*) was described using pressure (*p_l_*) and unstressed air volume in the larynx (*V_u,l_*).

(21) $$\frac{dp_{l}}{dt}=\frac{1}{C_{l}}\left(\frac{p_{atm}-p_{l}}{R_{airl}}-\frac{p_{l}-p_{tr}}{R_{ltr}}\right)$$

(22) $$Q_{airl}=\frac{p_{atm}-p_{l}}{R_{airl}}$$

(23) $$V_{l}=C_{l}p_{l}+V_{u,l}$$

Here, *C_l_*, *R_airl_* and *R_ltr_* represent compliance of the larynx, the resistance between mouth and larynx, and resistance between larynx and trachea, respectively. Air pressure, flow rate and volume in the other compartments of the respiratory system were described in a similar way using corresponding variables and parameter values.

### 2.4 Gas Exchange

The gas exchange in the lungs occurs between alveoli and blood in pulmonary capillaries. The inspired oxygen diffused from the alveoli to blood in pulmonary capillaries and the expired carbon dioxide diffused from blood in pulmonary capillaries to alveoli.^[^ ^37^ ^]^ The partial pressures of oxygen and carbon dioxide in the larynx (*p_l,i_*), trachea (*p_tr,i_*), bronchia (*p_b,i_*) and alveoli (*p_A,i_*) were given for inspiration in the equations below.

(24) $$\frac{dp_{l,i}}{dt}=\frac{1}{V_{l}}\left(Q_{airl}p_{atm,i}-Q_{ltr}p_{l,i}-p_{l,i}\frac{dV_{l}}{dt}\right)$$

(25) $$\frac{dp_{tr,i}}{dt}=\frac{1}{V_{tr}}\left(Q_{ltr}p_{l,i}-Q_{trb}p_{tr,i}-p_{tr,i}\frac{dV_{tr}}{dt}\right)$$

(26) $$\frac{dp_{b,i}}{dt}=\frac{1}{V_{b}}\left(Q_{trb}p_{tr,i}-Q_{bA}p_{b,i}-p_{b,i}\frac{dV_{b}}{dt}\right)$$

(27) $$\frac{dp_{A,i}}{dt}=\frac{1}{V_{A}}\left(Q_{bA}p_{b,i}-p_{A,i}\frac{dV_{A}}{dt}-D_{L,i}\left(p_{A,i}-p_{v,i}\right)V_{arp}\right)$$

The partial pressures of oxygen and carbon dioxide in the larynx, trachea, bronchia, and alveoli were given for expiration in the equations below.

(28) $$\frac{dp_{l,i}}{dt}=\frac{1}{V_{l}}\left(Q_{airl}p_{l,i}-Q_{ltr}p_{tr,i}-p_{l,i}\frac{dV_{l}}{dt}\right)$$

(29) $$\frac{dp_{tr,i}}{dt}=\frac{1}{V_{tr}}\left(Q_{ltr}p_{tr,i}-Q_{trb}p_{b,i}-p_{tr,i}\frac{dV_{tr}}{dt}\right)$$

(30) $$\frac{dp_{b,i}}{dt}=\frac{1}{V_{b}}\left(Q_{trb}p_{b,i}-Q_{bA}p_{A,i}-p_{b,i}\frac{dV_{b}}{dt}\right)$$

(31) $$\frac{dp_{A,i}}{dt}=\frac{1}{V_{A}}\left(Q_{bA}p_{A,i}-p_{A,i}\frac{dV_{A}}{dt}-D_{L,i}\left(p_{A,i}-p_{v,i}\right)V_{arp}\right)$$

Here, *i* represents gas species (O_2_ and CO_2_) in the airways. *p_atm,i_*, *p_v,i_*, *D_L,i_* and *V_arp_* denote the partial pressure of atmospheric O_2_ and CO_2_, the partial pressure of venous O_2_ and CO_2_, the diffusion capacity of O_2_ and CO_2_ and the volume of pulmonary peripheral vessels, respectively.

### 2.5 Pressures of Oxygen and Carbon Dioxide in Blood

The partial pressures of dissolved oxygen in arterial and venous blood (*P_a,O2_*, *P_v,O2_*) were modeled using dissolved oxygen contents in arterial and venous blood (*Sol_a,O2_*, *Sol_v,O2_*).

(32) $$p_{a,O_{2}}=\frac{Sol_{a,O_{2}}}{0.0031}$$

(33) $$p_{v,O_{2}}=\frac{Sol_{v,O_{2}}}{0.0031}$$

The contents of dissolved oxygen in arterial and venous blood were assumed constant. The partial pressure of the arterial and venous oxygen were corrected using blood temperature (*T*), pH of arterial and venous blood and the partial pressures of carbon dioxide in arterial and venous blood (*P_aCO2_*, *P_vCO2_*).^[^ ^38^ ^]^

(34) $$p_{a,O_{2}}=p_{a,O_{2}}\cdot 10^{0.024\cdot \left(37-T\right)+0.4\cdot \left(pH_{a}-7.4\right)}{\left(\frac{40}{p_{aCO_{2}}}\right)}^{0.06}$$

(35) $$p_{v,O_{2}}=p_{v,O_{2}}\cdot 10^{0.024\cdot \left(37-T\right)+0.4\cdot \left(pH_{v}-7.4\right)}{\left(\frac{40}{p_{vCO_{2}}}\right)}^{0.06}$$

Here, *pH_a_* and *pH*_v_ denote the *pH* value of arterial and venous blood, respectively. The partial pressure of carbon dioxide in arterial and venous blood models was adopted from Giovannini et al (1993).^[^ ^39^ ^]^ The partial pressures of carbon dioxide in arterial and venous blood were given in the equations below.

(36) $$\begin{array}{c} p_{aCO_{2}}=\frac{C_{p,aCO_{2}}-\left(pH-pH_{a}\right)\cdot \left(-18.2532-3.103044\cdot Hb\right)}{0.06868\cdot 10^{1.0424\cdot pH_{a}-6.410361}} \end{array}$$

(37) $$\begin{array}{c} p_{vCO_{2}}=\frac{C_{p,vCO_{2}}-\left(pH-pH_{v}\right)\cdot \left(-18.2532-3.103044\cdot Hb\right)}{0.06868\cdot 10^{1.0424\cdot pH_{v}-6.410361}} \end{array}$$

Here, *C_p,aCO2_* and *C_p,vCO2_* denote the carbon dioxide content of arterial and venous blood plasma. *Hb* is hemoglobin concentration and *pH* represent the reference value of blood *pH*. Carbon dioxide contents of the arterial and venous blood were described using plasma CO_2_ solubility (*s*), partial pressure of plasma CO_2_ in arterial and venous blood (*P_p,aCO2_* and *P_p,vCO2_*), apparent *pK* of the CO_2_‐bicarbonate system (*pK’*), and *pH* values of arterial and venous blood.^[^ ^40^ ^]^

(38) $$C_{p,aCO_{2}}=2.226\cdot s\cdot P_{p,aCO_{2}}\cdot \left(1+10^{pH_{a}-p{K^{\prime }}_{a}}\right)$$

(39) $$C_{p,vCO_{2}}=2.226\cdot s\cdot P_{p,vCO_{2}}\left(1+10^{pH_{v}-p{K^{\prime }}_{v}}\right)$$

Plasma CO_2_ solubility (*s*) was described using blood temperature (*T*).^[^ ^40^ ^]^

(40) $$s=0.0307+0.00057\cdot \left(37-T\right)+0.00002\cdot {\left(37-T\right)}^{2}$$

The relationship between the partial pressure of plasma CO_2_ and *pH* values of arterial and venous blood was defined by using data from Douglas et al. (1988).^[^ ^40^ ^]^

(41) $$P_{p,aCO_{2}}=-164.01\cdot pH_{a}+1250.7$$

(42) $$P_{p,vCO_{2}}=-164.01\cdot pH_{v}+1250.7$$

Apparent *pK* of the CO_2_‐bicarbonate system for arterial and venous blood (*pK^’^_a_* and *pK^’^_v_*) were defined using reference values of blood *pH*, arterial and venous blood *pH* (*pH*_a_ and *pH_v_*), and blood temperature (*T*).^[^ ^40^ ^]^

(43) $$\begin{array}{ccc} p{K^{\prime }}_{a} & = & 6.086+0.042\cdot \left(pH-pH_{a}\right)+\left(38-T\right)\cdot \\ & & \left(0.00472+0.00139\cdot \left(pH-pH_{a}\right)\right) \end{array}$$

(44) $$\begin{array}{ccc} p{K^{\prime }}_{v} & = & 6.086+0.042\cdot \left(pH-pH_{v}\right)+\left(38-T\right)\cdot \\ & & \left(0.00472+0.00139\cdot \left(pH-pH_{v}\right)\right) \end{array}$$

### 2.6 Oxygen Saturation of Arterial and Venous Blood

Arterial blood oxygen saturation (*S_aO2_*) was defined using the partial pressure of arterial oxygen (*P_aO2_*).

(45) $$\begin{array}{c} S_{aO_{2}}=\left({\left(\left({\left(P_{aO_{2}}^{3}+150\cdot P_{aO_{2}}\right)}^{-1}\cdot 23400\right)+1\right)}^{-1}\right)\cdot 100 \end{array}$$

Venous blood oxygen saturation (*S_vO2_*) was defined using the partial pressure of venous oxygen (*P_vO2_*).

(46) $$\begin{array}{c} S_{vO_{2}}=\left({\left(\left({\left(P_{vO_{2}}^{3}+150\cdot P_{vO_{2}}\right)}^{-1}\cdot 23400\right)+1\right)}^{-1}\right)\cdot 100 \end{array}$$

### 2.7 Sensitivity Analysis

**Figure 2:**
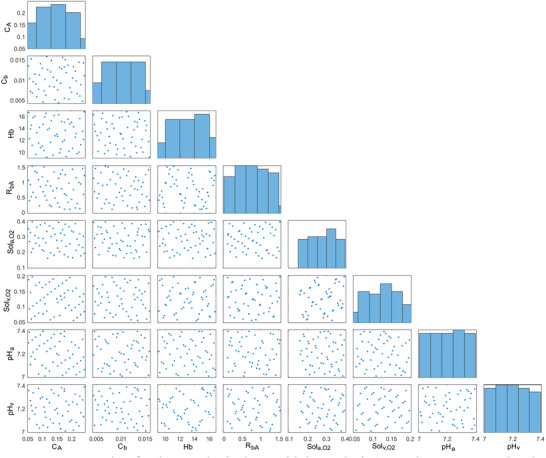
Scatter plots for the samples in the sensitivity analysis (C_A_ and C_b_, represent alveolar and bronchial compliances, Hb represents Hemoglobin, R_bA_ represents the resistance between bronchi and alveoli, Sol_a,O2_ and Sol_v,O2_ represent dissolved O_2_ content in arterial and venous blood, pH_a_ and pH_v_ represent arterial and venous blood pH).

Sensitivity analysis was performed to evaluate the effects of the modified parameters in the numerical model to simulate acidosis, COPD and anemia on the cerebral flow rate, cardiac output, mean arterial pressure, arterial and venous O_2_ saturation, partial pressures of CO_2_ and O_2_ in arterial and venous blood, partial pressure of CO_2_ and O_2_ in alveoli and alveolar volume. Fifty samples with uniform distributions were generated for each parameter in Simulink Sensitivity Analyzer. Scatter plots for the samples used in the sensitivity analysis are given in **Figure** **2**.

### 2.8 Simulation of Acidosis, COPD, and Anemia

The range of the arterial blood pH is between 7.35 and 7.45 for healthy subjects.^[^ ^13^ ^]^ The arterial and venous blood pH were 7.394 and 7.37 in the numerical model simulating a healthy condition. The arterial blood pH is lower in patients with COPD than in healthy subjects.^[^ ^13^ ^]^ The arterial and venous pH were tuned as 7.37 and 7.344 in the numerical model simulating COPD.

The outdoor CO_2_ level is 400 ppm in the present days whereas it may be increased around to 2000 ppm by 2250.^[^ ^41^ ^]^ As a result, the average outdoor temperature may be 11 °C higher than present day.^[^ ^41^ ^]^ Also, the increase in outdoor temperature may cause kidney failure.^[^ ^42^, ^43^ ^]^ The blood pH is lower in patients with kidney failure.^[^ ^44^ ^]^ The arterial blood pH may reduce further in patients with COPD.^[^ ^45^ ^]^ The arterial and venous blood pH values were decreased to 7.2 and 7.176 respectively in the models simulating acidosis and COPD.

The small airways constrict in COPD patients.^[^ ^46^ ^]^ Also, lung elastance was higher in COPD patients than in healthy subjects.^[^ ^47^ ^]^ The resistance between bronchi and alveoli (*R_bA_*) was increased from 0.1 to 1 cmH_2_Os/L and compliances of bronchi (*C_b_*) and alveoli (*C_A_*) were decreased from 0.0133 to 0.0067 L/cmH_2_O and from 0.175 to 0.0875 L/cmH_2_O to simulate COPD.

Hemoglobin concentration is around 15 g dL^−1^ in non‐anemic conditions.^[^ ^48^ ^]^ The threshold of hemoglobin level for anemia is 13 g dL^−1^ whereas World Health Organisation defines the threshold of hemoglobin level for females as 12 g dL^−1^.^[^ ^48^ ^]^ The hemoglobin concentration was decreased from 15 to 11 g dL^−1^ to simulate COPD with anemia in the model.

The partial pressure of oxygen in arterial blood is around 62 mmHg in patients with COPD.^[^ ^49^, ^50^ ^]^ The partial pressure of oxygen in venous blood is lower than the partial pressure of oxygen in arterial blood.^[^ ^37^ ^]^ Dissolved oxygen contents in arterial and venous blood (*Sol_aO2_* and *Sol_vO2_*) were decreased from 0.31 to 0.20 mL dL^−1^ and from 0.13 to 0.084 mL dL^−1^ respectively to tune the partial pressure of arterial and venous blood in the model simulating COPD.

## 3 Results

**Figure 3:**
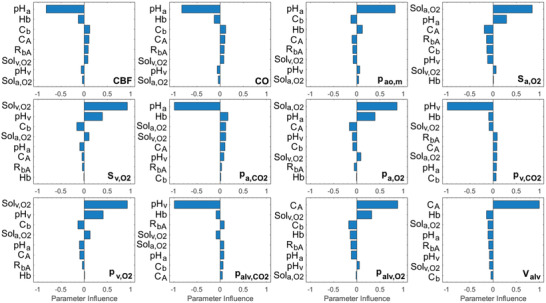
Parameter influence on variables evaluated for COPD and anemia. Hb, pH_a_, pH_v_, C_A_, C_b_, R_bA_, Sol_a,O2_ and Sol_v,O2_ represent hemoglobin, arterial and venous blood pH, alveolar and bronchial compliances, resistance between bronchi and alveoli and dissolved O_2_ content in arterial and venous blood (CBF: Cerebral Blood Flow Rate, CO: Cardiac output, p_ao,m_: Mean arterial pressure, S_a,O2_: Arterial O_2_ saturation, S_v,O2_: Venous O_2_ saturation, p_a,CO2_: The partial pressure of CO_2_ in arterial blood, p_a,O2_: The partial pressure of O_2_ in arterial blood, p_v,CO2_: The partial pressure of CO_2_ in venous blood, p_v,O2_: The partial pressure of O_2_ in venous blood, p_alv,CO2_: The partial pressure of CO_2_ in alveoli, p_alv,O2_: The partial pressure of O_2_ in alveoli and V_alv_: Alveolar volume).

The bar charts showing the parameter influence on the variables evaluated in the numerical model are given in **Figure** **3**.

**Figure 4:**
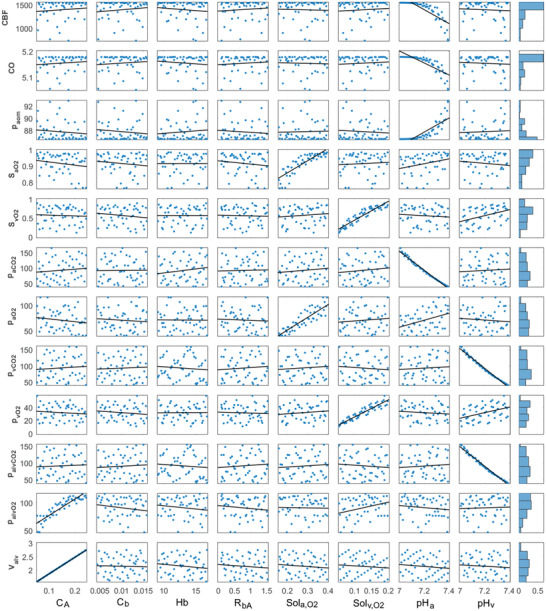
Scatter plots with linear lines in the sample scatter plot for variables evaluated in the sensitivity analysis for the models simulating chronic obstructive pulmonary disease with and without anemia. Hb, pH_a_, pH_v_, C_A_, C_b_, R_bA_, Sol_aO2_ and Sol_vO2_ represent hemoglobin, arterial and venous blood pH, alveolar and bronchial compliances, the resistance between bronchi and alveoli and dissolved O_2_ content in arterial and venous blood (CBF: Cerebral Blood Flow Rate, CO: Cardiac output, p_ao,m_: Mean arterial pressure, S_a,O2_: Arterial O_2_ saturation, S_v,O2_: Venous O_2_ saturation, p_a,CO2_: The partial pressure of CO_2_ in arterial blood, p_a,O2_: The partial pressure of O_2_ in arterial blood, p_v,CO2_: The partial pressure of CO_2_ in venous blood, p_v,O2_: The partial pressure of O_2_ in venous blood, p_alv,CO2_: The partial pressure of CO_2_ in alveoli, p_alv,O2_: The partial pressure of O_2_ in alveoli and V_alv_: Alveolar volume).

Arterial blood pH (pH_a_) was the most influential parameter on cerebral blood flow rate, cardiac output, mean aortic pressure and the partial pressure of CO_2_ in arterial blood. Also, there was an inverse correlation between Arterial blood pH (pH_a_) and the partial pressure of CO_2_ in arterial blood (p_a,CO2_). Venous blood pH (pH_v_) was the most influential parameter on the partial pressure of CO_2_ in venous blood (p_v,CO2_) and alveoli (p_alv,CO2_). Dissolved arterial O_2_ content (Sol_a,O2_) was the most influential parameter on the arterial O_2_ saturation (S_aO2_) and the partial pressure of O_2_ in arterial blood (p_a,O2_) whereas dissolved venous O_2_ content (Sol_v,O2_) was the most influential parameter on the venous O_2_ saturation (S_v,O2_) and the partial pressure of O_2_ in venous blood (p_v,O2_). Linear fit curves in the sample plots in the sensitivity analysis for the evaluated parameters in the numerical model are given in **Figure** **4**.

**Figure 5:**
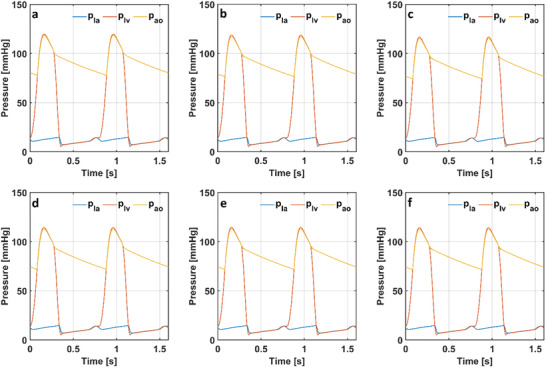
Hemodynamic pressures in the numerical models simulating a) a healthy condition, b) COPD, c) COPD with anemia, d) respiratory acidosis, e) respiratory acidosis and COPD f) respiratory acidosis and COPD with anemia. (la: left atrium, lv: left ventricle and ao: aorta).

The cerebral blood flow rate, cardiac output and mean arterial pressure were slightly sensitive to alveolar compliance (C_A_), bronchial compliance (C_b_), hemoglobin (Hb), the resistance between bronchia and alveoli (R_bA_), dissolved arterial O_2_ content (Sol_a,O2_), dissolved venous O_2_ content (Sol_v,O2_) and venous blood pH (pH_v_) whereas they were remarkably sensitive to arterial blood pH (pH_a_). Arterial O_2_ saturation (S_aO2_) was remarkably sensitive to dissolved arterial O_2_ content (Sol_a,O2_) whereas venous O_2_ saturation (S_v,O2_) was remarkably sensitive to dissolved venous O_2_ (Sol_v,O2_). The partial pressures of CO_2_ in arterial and venous blood (p_aO2_ and p_vO2_) were highly sensitive to arterial blood pH (pH_a_) and venous blood pH (pH_v_), whereas slightly sensitive to the other parameters. The partial pressure of arterial O_2_ (p_aO2_) was highly sensitive to dissolved arterial O_2_ content (Sol_a,O2_). The partial pressure of venous O_2_ (p_v,O2_) was highly sensitive to dissolved venous O_2_ content (V_vO2_). The partial pressure of alveolar CO_2_ (p_A,CO2_) was highly sensitive to venous blood pH (pH_v_). The partial pressure of alveolar O_2_ (p_A,O2_) and alveolar volume (V_alv_) were highly sensitive to alveolar compliance (C_A_). Also, the partial pressure of alveolar O_2_ (p_A,O2_) was sensitive to dissolved venous O_2_ (Sol_v,O2_). The left atrial and ventricular and aortic pressures (p_la_, p_lv_ and p_ao_) in the models simulating healthy conditions, COPD with and without anemia and respiratory acidosis are given in **Figure** **5**.

The maximal left ventricular pressure (p_lv_) was 120 mmHg in the numerical model simulating a healthy condition. The maximal left ventricular pressures (p_lv_) were 119 and 117 mmHg in the models simulating COPD with and without anemia, respectively. The maximal left ventricular pressure (p_lv_) decreased to 114 mmHg in the models simulating respiratory acidosis, respiratory acidosis and COPD with and without anemia.

**Figure 6:**
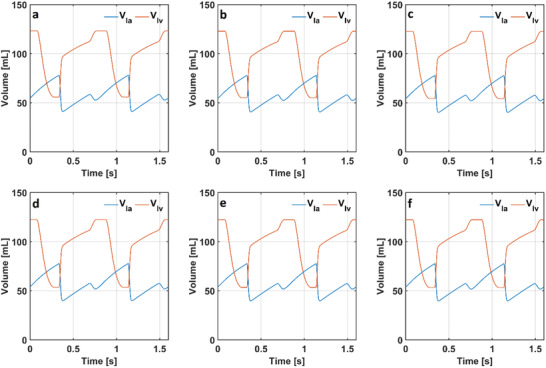
Hemodynamic volumes in the models simulating a) a healthy condition, b) COPD, c) COPD with anemia, d) respiratory acidosis, e) respiratory acidosis and COPD, f) respiratory acidosis and COPD with anemia. (la: left atrium and lv: left ventricle).

The aortic pressure (p_ao_) changed between 78 and 118 mmHg in the numerical model simulating a healthy condition. The aortic pressures (p_ao_) changed between 76 mmHg and 117 in the numerical model simulating COPD whereas it changed between 74 and 115 mmHg in the model simulating COPD with anemia. The range of the aortic pressure (p_ao_) slightly decreased in conditions of respiratory acidosis. The aortic pressure (p_ao_) changed between 72 and 113 mmHg in the numerical model simulating respiratory acidosis and respiratory acidosis and COPD. The aortic pressure (p_ao_) changed between 71 and 113 mmHg in the model simulating respiratory acidosis and COPD with anemia. The left atrial and ventricular volumes in the models simulating a healthy condition, COPD with and without anemia and respiratory acidosis are given in **Figure** **6**.

**Figure 7:**
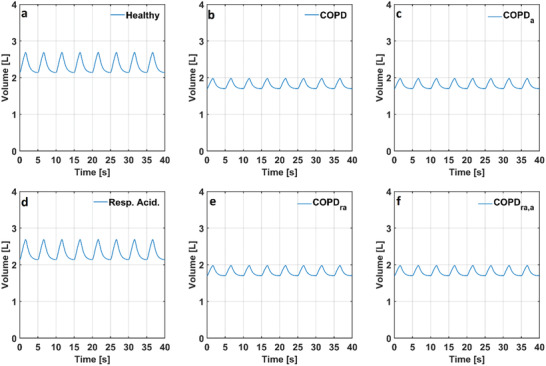
Alveolar volumes (V_A_) in the numerical models simulating a) a healthy condition, b) COPD, c) COPD with anemia, d) respiratory acidosis e) respiratory acidosis and COPD f) respiratory acidosis and COPD with anemia. (COPD: Chronic obstructive pulmonary disease, COPD_a_: Chronic obstructive pulmonary disease with anemia, Resp. Acid.: Respiratory Acidosis, COPD_ra_: Chronic obstructive pulmonary disease with respiratory acidosis, COPD_ra,a_: Chronic obstructive pulmonary disease with respiratory acidosis and anemia).

Left ventricular volume (V_lv_) changed between 56 and 123 mL in the numerical model simulating a healthy condition. Left ventricular volume (V_lv_) changed between 55 and 123 mL in the models simulating COPD with and without anemia. There was no noticeable change in the left ventricular volume (V_lv_) in the numerical model simulating respiratory acidosis. Left ventricular volume (V_lv_) changed between 56 and 122 mL in the model simulating respiratory acidosis, whereas it changed between 53 and 122 mL in the model simulating respiratory acidosis and COPD with and without anemia. The alveolar volumes (V_A_) in the numerical models simulating a healthy condition, COPD with and without anemia and respiratory acidosis are given in **Figure** **7**.

**Figure 8:**
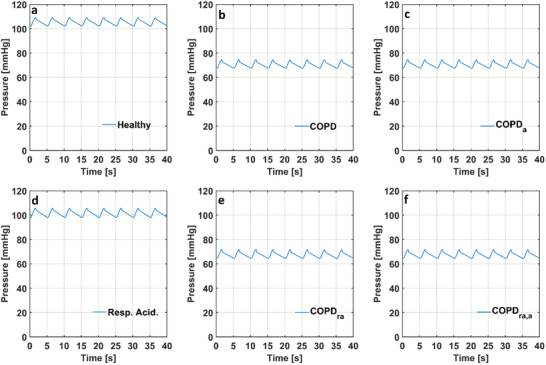
The partial pressure of O_2_ in alveoli (p_A,O2_) in the numerical models simulating a) a healthy condition, b) COPD, c) COPD with anemia, d) respiratory acidosis, e) respiratory acidosis and COPD f) respiratory acidosis and COPD with anemia. (COPD: Chronic obstructive pulmonary disease, COPD_a_: Chronic obstructive pulmonary disease with anemia, Resp. Acid.: Respiratory Acidosis, COPD_ra_: Chronic obstructive pulmonary disease with respiratory acidosis, COPD_ra,a_: Chronic obstructive pulmonary disease with respiratory acidosis and anemia).

The decrease in the alveolar compliance (C_A_) caused alveolar volume (V_A_) to decrease in the models simulating COPD with and without anemia. Alveolar volume (V_A_) changed between 2.14 and 2.69 L in the numerical model simulating a healthy condition whereas the alveolar volume changed between 1.70 and 1.98 L in the models simulating COPD with and without anemia. Also, the simulation results showed that respiratory acidosis did not affect the alveolar volume (V_A_). The partial pressure of O_2_ in alveoli (p_A,O2_) in the models simulating a healthy condition, COPD with and without anemia and respiratory acidosis are given in **Figure** **8**.

**Figure 9:**
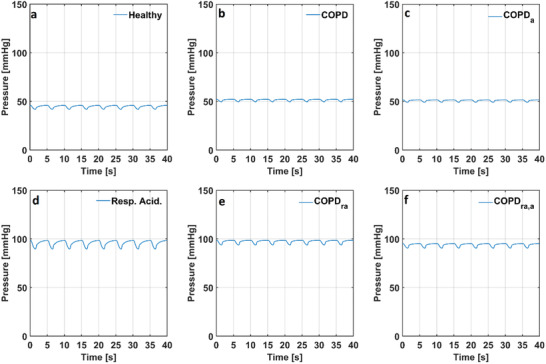
The partial pressure of CO_2_ in alveoli (p_ACO2_) in the models simulating a) a healthy condition, b) COPD, c) COPD with anemia, d) respiratory acidosis, e) respiratory acidosis and COPD, f) respiratory acidosis and COPD with anemia. (COPD: Chronic obstructive pulmonary disease, COPD_a_: Chronic obstructive pulmonary disease with anemia, Resp. Acid.: Respiratory Acidosis, COPD_ra_: Chronic obstructive pulmonary disease with respiratory acidosis, COPD_ra,a_: Chronic obstructive pulmonary disease with respiratory acidosis and anemia).

The partial pressure of O_2_ in alveoli (p_A,O2_) changed between 102 and 109 mmHg in the numerical model simulating a healthy condition. The partial pressure of O_2_ in alveoli (p_A,O2_) decreased in the models simulating COPD with and without anemia. The partial pressure of O_2_ in alveoli (p_A,O2_) changed between 67 and 75 mmHg in the models simulating COPD with and without anemia. Also, the partial pressure of O_2_ in alveoli (p_A,O2_) slightly decreased in the models simulating respiratory acidosis and respiratory acidosis and COPD with and without anemia. The partial pressure of CO_2_ in alveoli (p_A,CO2_) in the models simulating healthy a condition, COPD with and without anemia and respiratory acidosis are given in **Figure** **9**.

**Figure 10:**
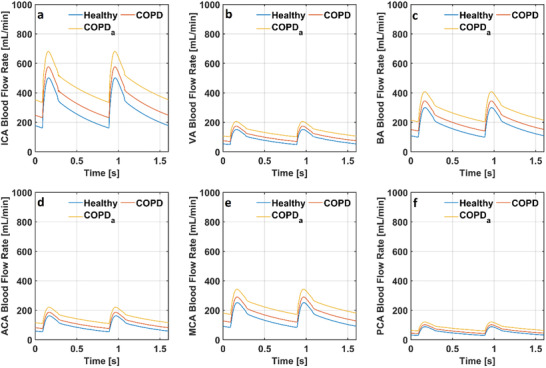
Cerebral blood flow rate in the models simulating healthy condition and COPD with and without anemia through a) internal carotid arteries b) vertebral arteries c) basilar artery d) anterior cerebral arteries e) middle cerebral arteries and f) posterior cerebral arteries (COPD: Chronic obstructive pulmonary disease, COPD_a_: Chronic obstructive pulmonary disease with anemia).

The partial pressure of CO_2_ in alveoli (p_A,CO2_) changed between 42 and 46 mmHg in the numerical model simulating a healthy condition. The partial pressure of CO_2_ in alveoli (p_A,CO2_) was higher in the models simulating COPD with and without anemia due to lower blood pH and it changed between 49 and 52 mmHg. The partial pressure of CO_2_ in alveoli (p_A,CO2_) changed between 89 and 98 mmHg in the model simulating respiratory acidosis, whereas it changed between 94 and 98 mmHg in the model simulating respiratory acidosis and COPD. The partial pressure of CO_2_ in alveoli (p_A,CO2_) slightly decreased in the model simulating respiratory acidosis and COPD with anemia. The blood flow rate through internal carotid arteries (ICA), vertebral arteries (VA), basilar artery (BA), anterior cerebral arteries (ACA), middle cerebral arteries (MCA) and posterior cerebral arteries (PCA) in the models simulating a healthy condition and COPD with and without anemia are given in **Figure** **10**.

**Figure 11:**
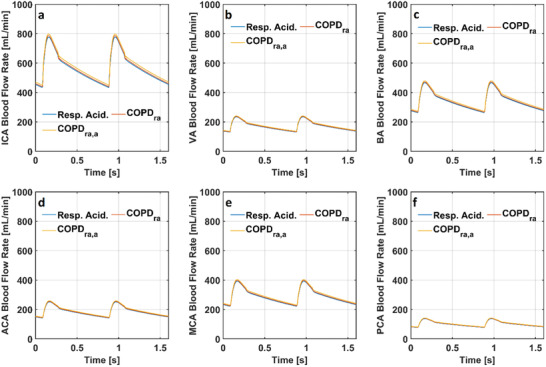
Cerebral blood flow rate in the models simulating respiratory acidosis and respiratory acidosis and COPD with and without anemia through a) internal carotid arteries b) vertebral arteries c) basilar artery d) anterior cerebral arteries e) middle cerebral arteries and f) posterior cerebral arteries (COPD_ra_: Chronic obstructive pulmonary disease with respiratory acidosis, COPD_ra,a_: Chronic obstructive pulmonary disease with respiratory acidosis and anemia and Resp. Acid.: Respiratory Acidosis).

In the numerical model simulating a healthy condition, the blood flow rates through internal carotid arteries (ICA) changed between 161 and 502 mL min^−1^. The blood flow rate through vertebral arteries (VA) changed between 49 and 151 mL min^−1^. The blood flow rate through the basilar artery (BA) changed between 99 and 300 mL min^−1^. The blood flow rate through anterior cerebral arteries (ACA) changed between 53 and 162 mL min^−1^. The blood flow rate through middle cerebral arteries (MCA) changed between 83  and 252 mL min^−1^, whereas the blood flow rate through posterior cerebral arteries (PCA) changed between 29 and 89 mL min^−1^. The cerebral blood flow rates through these arteries were higher in the model simulating COPD than in the numerical model simulating a healthy condition due to higher partial pressure of arterial CO_2_ (p_a,CO2_). Moreover, decreased hemoglobin (Hb) increased the blood flow rate in the cerebral arteries further because of reduced arterial O_2_ content (C_a,O2_) in the numerical model simulating COPD with anemia. The blood flow rate through internal carotid arteries (ICA), vertebral arteries (VA), basilar artery (BA), anterior cerebral arteries (ACA), middle cerebral arteries (MCA) and posterior cerebral arteries (PCA) in the models simulating respiratory acidosis and respiratory acidosis and COPD with and without anemia are given in **Figure** **11**.

In the model simulating respiratory acidosis, the blood flow rate through internal carotid arteries (ICA) changed between 434 and 777 mL min^−1^. The blood flow rate through vertebral arteries (VA) changed between 131 and 234 mL min^−1^. The blood flow rate through basilar artery (BA) changed between 263 and 466 mL min^−1^. The blood flow rate through anterior cerebral arteries (ACA) changed between 142 and 252 mL min^−1^. The blood flow rate through middle cerebral arteries (MCA) changed between 222 and 392 mL min^−1^, whereas the blood flow rate through posterior cerebral arteries (PCA) changed between 78 and 138 mL min^−1^. Cerebral blood flow rates through the cerebral arteries slightly increased in the models simulating respiratory acidosis and COPD with and without anemia.

Mean arterial pressure (MAP), cardiac output (CO), the partial pressures of arterial and venous O_2_ (p_a,O2_, p_v,O2_), partial pressures of arterial and venous CO_2_ (p_a,CO2_ and p_v,CO2_), total cerebral blood flow rate (CBF), lower body blood flow rate (LBBF), the mean blood flow rates through internal carotid arteries (ICA), vertebral arteries (VA), basilar artery (BA), anterior cerebral arteries (ACA), middle cerebral arteries (MCA) and posterior cerebral arteries (PCA), tidal volumes (TV), and arterial and venous blood O_2_ saturations (S_a,O2_, S_v,O2_) are given in **Table** **1** the models simulating a healthy condition, COPD with and without anemia, respiratory acidosis and respiratory acidosis and COPD with and without anemia.

**Table 1:** The results for arterial and venous blood gas pressure, mean cerebral blood flow rates and arterial and venous O_2_ saturation for the simulated physiological conditions (MAP: Mean arterial pressure, CO: Cardiac Output, COPD: Chronic obstructive pulmonary disease, LBBF: Lower Body Blood Flow Rate, ICA: Internal Carotid Arteries, VA: Vertebral Arteries, BA: Basilar Artery, ACA: Anterior Cerebral Arteries, MCA: Middle Cerebral Arteries, PCA: Posterior Cerebral Arteries and TV: Tidal Volume).

	Healthy	COPD	COPD with anemia	Respiratory Acidosis	Respiratory Acidosis and COPD	Respiratory Acidosis and COPD with Anemia
MAP [mmHg]	93	92	90	87	87	87
CO [L min^−1^]	5.05	5.085	5.128	5.166	5.175	5.180
P_aO2_ [mmHg]	99	62	62	79	51	51
P_vO2_ [mmHg]	40	25	25	32	21	21
P_aCO2_ [mmHg]	40	46	46	91	91	88
P_vCO2_ [mmHg]	46	52	52	99	99	95
CBF [mL min^−1^]	737	923	1198	1473	1491	1524
LBBF [L min^−1^]	4.313	4.162	3.930	3.693	3.684	3.656
ICA [mL min^−1^]	283	355	460	566	573	585
VA [mL min^−1^]	85	107	139	171	173	177
BA [mL min^−1^]	171	214	278	342	346	353
ACA [mL min^−1^]	92	116	150	185	187	191
MCA [mL min^−1^]	144	180	234	288	291	298
PCA [mL min^−1^]	50	63	82	101	102	104
TV [mL]	470	246	246	470	246	246
SaO_2_ [%]	98	91	91	96	86	86
SvO_2_ [%]	75	46	46	62	34	34

The mean arterial pressure (MAP) slightly decreased, whereas cardiac output (CO) slightly increased in the models simulating chronic obstructive pulmonary disease with and without anemia and respiratory acidosis conditions. The partial pressure of arterial O_2_ (p_a,O2_) decreased from 99 to 79 mmHg and the partial pressure of venous O_2_ (p_v,O2_) decreased from 40 to 32 mmHg in the respiratory acidosis. Also, the partial pressures of the arterial and venous O_2_ (p_aO2_ and p_vO2_) were lower in the models simulating chronic obstructive pulmonary disease with and without anemia and respiratory acidosis conditions. The partial pressure of the arterial CO_2_ (p_a,CO2_) increased from 40 to 91 mmHg, whereas the partial pressure of venous CO_2_ (p_v,CO2_) increased from 46 to 99 mmHg in respiratory acidosis. The partial pressures of the arterial and venous CO_2_ (p_a,CO2_ and p_v,CO2_) were higher in the models simulating chronic obstructive pulmonary disease with and without anemia and respiratory acidosis. Tidal volumes (TV) were higher in the models simulating a healthy condition and respiratory acidosis than in the models simulating respiratory acidosis and COPD with and without anemia. Arterial and venous blood O_2_ saturations (S_a,O2_, S_v,O2_) were decreased remarkably in the models simulating COPD with and without anemia and slightly decreased in the models with respiratory acidosis. However, the results showed that anemia did not change arterial and venous blood O_2_ saturations (S_a,O2_, S_v,O2_).

## 4 Discussion

In this study, a numerical model including the cardiovascular system, cerebral circulation, baroreflex and cerebral blood flow rate autoregulatory mechanisms, respiratory system, and blood gas contents was used to evaluate the effect of respiratory acidosis, COPD, and anemia on the blood flow.

The ranges of systolic and diastolic blood pressures in the aorta for healthy physiological conditions change between 90–140 and 60–90 mmHg, respectively.^[^ ^51^ ^]^ The systolic and diastolic blood pressures in the aorta are within physiological ranges for patients with COPD.^[^ ^52^ ^]^ Mean arterial blood pressure decreases by 7.5% with a decrease in the arterial blood pH (pH_a_) to 7.09.^[^ ^53^ ^]^ In this study, arterial blood pH (pH_a_) was decreased to 7.2, and mean arterial pressure decreased by 6.5%. The simulation results were within physiological range for the simulated healthy condition, respiratory acidosis, and COPD with and without anemia and respiratory acidosis. Also, there were minor differences among left ventricular volumes (V_lv_) in the models simulating the healthy condition, respiratory acidosis, and COPD with and without anemia and respiratory acidosis.

The increase in resistance of respiratory airways due to bronchoconstriction may cause alveolar ventilation to decrease.^[^ ^54^ ^]^ The resistance and elastance of respiratory airways are also higher in patients with COPD than in healthy subjects.^[^ ^45^, ^47^ ^]^ Therefore, higher resistance between bronchi and alveoli (R_bA_) and lower compliances in bronchi and alveoli (C_b_ and C_A_) were used to simulate COPD with and without anemia and respiratory acidosis. Also, the decrease in alveolar volume (V_A_) is associated with an increase in the resistance of respiratory airways.^[^ ^54^ ^]^ Higher respiratory airway resistance and lower compliances caused alveolar volume (V_A_) and tidal volume (TV) to decrease. The increase in resistance and decrease in compliance of respiratory airways may cause shortness of breath during daily activities.

The partial pressures of alveolar O_2_ and CO_2_ (p_A,O2_ and p_A,CO2_) depend on the partial pressures of O_2_ and CO_2_ in venous blood (p_v,O2_ and p_v,CO2_) in the numerical model. The partial pressure of O_2_ in blood was lower in the numerical model simulating COPD with anemia. Therefore, the partial pressure of alveolar O_2_ (p_A,O2_) was lower in COPD with and without anemia than in the simulated healthy condition. Studies about climate change predict that atmospheric O_2_ levels in the atmosphere will not change remarkably.^[^ ^55^ ^]^ Therefore, the partial pressure of the alveolar O_2_ (p_A,O2_) level in healthy conditions will not be affected by climate change. The partial pressure of alveolar CO_2_ (p_A,CO2_) was higher in the models simulating COPD with and without anemia than the numerical model simulating a healthy condition. Also, the partial pressure of arterial CO_2_ (p_a,CO2_) was remarkably higher in the models simulating respiratory acidosis and COPD with and without anemia and respiratory acidosis. The partial pressure of arterial CO_2_ (p_a,CO2_) depends on the produced, eliminated, and inspired CO_2_.^[^ ^14^ ^]^ The elevated outdoor CO_2_ due to climate change may cause inspired CO_2_ to increase. The level of indoor CO_2_ depends on the outdoor CO_2_ level and it may be six to ten times higher than the level of outdoor CO_2_ especially in crowded areas such as offices and classrooms.^[^ ^5^, ^56^ ^]^ By the year 2250, the level of inspired CO_2_ may be higher than 10 000 ppm in crowded indoor areas. Thus, the partial pressure of arterial CO_2_ (p_a,CO2_) may increase due to climate change. The increase in the partial pressure of arterial CO_2_ (p_a,CO2_) causes acidosis to increase and decrease in blood pH.^[^ ^57^ ^]^ Although kidneys regulate the blood pH to decrease acidosis, the increase in temperature causes acute kidney injury.^[^ ^42^, ^43^ ^]^ Also, the climate models show that atmospheric temperature in 2250 may be about 11 °C higher than the present day.^[^ ^41^ ^]^ Kidney failure may increase and pH in the blood cannot be regulated by the kidneys. The partial pressure of arterial CO_2_ (p_a,CO2_) is higher in patients with COPD than in healthy subjects due to lower blood pH.^[^ ^45^, ^58^ ^]^ Also, the partial pressure of venous CO_2_ (p_v,CO2_) is higher than the partial pressure of arterial CO_2_ (p_aCO2_).^[^ ^59^ ^]^ The decrease in blood pH caused the partial pressure of arterial CO_2_ (p_a,CO2_) to increase both in the models simulating a healthy condition, respiratory acidosis, and chronic obstructive pulmonary disease with and without anemia and respiratory acidosis. The simulation results were in line with clinical data.^[^ ^13^, ^45^, ^58^ ^]^

An increase in the breathing CO_2_ may cause cognitive performance to worsen.^[^ ^4^, ^5^, ^56^, ^60^ ^]^ Also, elevated CO_2_ may cause sick‐building syndrome.^[^ ^56^ ^]^ The increase in the partial pressure of arterial CO_2_ (p_a,CO2_) leads to an increase in cerebral blood flow rate.^[^ ^20^ ^]^ The partial pressures of arterial and venous CO_2_ (p_a,CO2_ and p_v,CO2_) were defined using blood pH in the models. Thus, the decrease in the blood pH caused the partial pressure of CO_2_ in the blood to increase. Also, the decrease in the arterial O_2_ content (C_a,O2_) leads to an increase in cerebral blood flow rate.^[^ ^61^, ^62^ ^]^ The arterial O_2_ content (C_a,O2_) was described using hemoglobin (Hb) in the blood. The decrease in hemoglobin (Hb) led to a decrease in arterial O_2_ content (C_a,O2_) and an increase in cerebral blood flow rate in the simulations. The increase in cerebral blood flow rate may cause intracranial hemorrhage and stroke^[^ ^22^, ^63^ ^]^. The simulation results showed that the cerebral blood flow rate was higher in the model simulating respiratory acidosis, COPD with and without anemia and respiratory acidosis than the numerical model simulating a healthy condition. The increase in cerebral blood flow rate led to a decrease lower body blood flow rate (LBBF) in both models simulating respiratory acidosis and chronic obstructive pulmonary disease with and without anemia and respiratory acidosis. The decrease in lower body blood flow rate (LBBF) may cause numbness and tingling in the lower limb of the body. Also, kidney diseases may increase due to a decrease in lower body blood flow rate in respiratory acidosis and COPD with and without anemia and respiratory disease.

Arterial oxygen saturation (S_a,O2_) was lower in the model simulating COPD with and without anemia than in the model simulating a healthy condition. Therefore, exacerbation may occur in respiratory diseases. Also, the results showed that respiratory acidosis caused arterial O_2_ saturation (S_a,O2_) to decrease to below 90%. The results support that morbidity and mortality may increase due to climate change in patients with COPD and anemia. Also, rate of the mortality may increase in patients with COPD and respiratory acidosis.

## 5 Conclusion

In this study, numerical modeling was used to evaluate the effects of acidosis which may be a result of increased CO_2_ levels and temperatures on cardiovascular and respiratory system functions and cerebral blood flow rates for a healthy condition, COPD and anemia. The simulation results showed that although the cardiovascular function was not significantly altered, the respiratory function and cerebral blood flow rates were altered remarkably.

Quantification of physiological parameters due to the effects of global warming may help to evaluate and predict the effects of climate change on physiological systems and human health and take measures accordingly.

## Conflict of Interest

The authors declare no conflict of interest.

## Data Availability

The data that support the findings of this study are available from the corresponding author upon reasonable request.
